# Current Methods for the Isolation and Cultivation of Microglia (Review)

**DOI:** 10.17691/stm2021.13.6.10

**Published:** 2021-12-28

**Authors:** N.A. Malinovskaya, O.V. Frolova, K.O. Shishelova, Yu.A. Panina

**Affiliations:** Professor, Department of Biological Chemistry with Courses in Medical, Pharmaceutical and Toxicological Chemistry; Krasnoyarsk State Medical University named after Professor V. F. Voino-Yasenetsky, 1 Partizana Zheleznyaka St., Krasnoyarsk, 660022, Russia; Senior Laboratory Assistant, Research Institute of Molecular Medicine and Pathobiochemistry; Krasnoyarsk State Medical University named after Professor V. F. Voino-Yasenetsky, 1 Partizana Zheleznyaka St., Krasnoyarsk, 660022, Russia; PhD Student, Department of Biological Chemistry with Courses in Medical, Pharmaceutical and Toxicological Chemistry; Krasnoyarsk State Medical University named after Professor V. F. Voino-Yasenetsky, 1 Partizana Zheleznyaka St., Krasnoyarsk, 660022, Russia; Associate Professor, Department of Biological Chemistry with Courses in Medical, Pharmaceutical and Toxicological Chemistry; Krasnoyarsk State Medical University named after Professor V. F. Voino-Yasenetsky, 1 Partizana Zheleznyaka St., Krasnoyarsk, 660022, Russia

**Keywords:** microglia, microglia morphotypes, neurodegeneration, density gradient, 2D and 3D cultures, scaffolds, slice cultures, co-cultures

## Abstract

The role and morphological features of microglia (M1 and M2 microglia, “stellate”, “amoeboid”, giant, round-shaped, rod-shaped, dysfunctional, etc.) *in vivo* under physiological conditions and during the development of neurodegenerative diseases have been described. Various methods and techniques of microglia isolation from adult (density gradient isolation, use of “magnetic beads”, from mesenchymal bone marrow progenitor cells) and newborn (obtaining from a mixed glial culture, density gradient isolation) animals have been considered, including microglia isolation from the cerebral cortex or hippocampus. Various methods of cell cultivation have been shown, including obtaining two-dimensional and three-dimensional cell cultures (on scaffolds, hydrogels, nanofibers), co-cultures on slice cultures of the hippocampus, as well as changes in microglia during cultivation.

## Introduction

Microglia are the primary resident immune cells and the main cells of the neuroimmune system of the brain involved in the development, normal functioning, aging, and damage to the nervous system cells [1]. Microglial cells were discovered as early as 1918 by the Spanish neurobiologist P. Río Hortega, who developed a method for staining microglia and described their difference from other cells of the central nervous system [2]. However, for a long time, microglial cells did not attract the researchers’ attention until G.W. Kreutzberg’s group described the process of microglial activation in case of facial nerve damage in 1968 [3]. New methods for microglia isolation (for example, using density gradient isolation or “magnetic beads”) made it possible to perform a comparative genomic, transcriptome, and proteome analysis in adult organisms and during embryonic development, and obtain a line of mice with GFP-labeled microglia made it possible to visualize changes in their cells *in vivo* using two-photon microscopy [1], which contributed to the revision of the views on microglia functioning.

Microglia are cells of mesodermal origin (mesenchymal and myeloid), which, depending on the brain region, constitute from 5 to 12% of the cells of the central nervous system [1, 4]. Until recently, only the CD11b, CX3CR1, Iba1, F4/80 antigens were microglia markers. Thanks to RNA sequencing, new microglia-specific markers were revealed: S100A8, S100A9, HEXB, TMEM119, GPR34, P2RY12, Siglec-H, TREM2, OLFML3 [1].

Microglial cells are derived from the pool of primitive yolk sac macrophages. They are generated during embryonic development: in mice embryos — on the 8.5^th^ day, in humans — on the 13^th^ week of pregnancy, while ramified microglia develops on the 21^st^ week. “Amoeboid” cells are first found in the neuroepithelium approximately on the 8.5–9^th^ day [3].

Survival and maintenance of microglial proliferation depend on cytokines (for example, CSF1 and IL-34) and transcription factors (for example, IRF8). According to some controversial data [1, 3], with a deficiency of microglia in adults, the population of these cells is replenished by mesenchymal stem cells from the bone marrow, particularly, in pathology. The proliferation of nestin-positive brain cells and then their differentiation into microglia has also been reported [3]. Moreover, reprogramming of monocytes into microglia-like cells is possible (depending on the microenvironment of monocytes) [1, 3].

Microglial cells in humans are very heterogeneous in morphology, expression of the receptors for neurotransmitters (GABA, dopamine, serotonin, histamine, nicotine, neurotensins, galanin, endothelin, somatostatin, substance P, vasopressin), TLR4 signal transduction, macrophage inflammatory protein (MIP-1α) release, CD80 and CD86 expression. Besides, the differences in microglia which depend on the patients’ age and brain region have been found [3].

Currently, the impact on the functions of glial cells is an underestimated therapeutic strategy in neurodegeneration [5]. Many authors [6–10] consider microglia as a “double-edged blade” exhibiting both neuroprotective and neurodestructive properties. They play a role in the pathogenesis of various diseases, and in some cases have neuroprotective effects. Microglia are also a promising target for the correction of neurodegeneration [6–8]. For example, an impact on the phagocytic function of microglia has been proposed as a therapeutic strategy for the diseases of the central nervous system [9]. It has been shown to be promising to block the chemokine ligand/receptor-1 (CX3CL1/CX3CR1) or its signaling pathways in order to activate the M2 phenotype of microglia and reduce the expansion of the M1 phenotype of microglia. This leads to a decrease in neurological deficit during ischemia, specifically, to reperfusion, as shown in CX3CR1 knockout mice [10]. Therefore, it is very relevant to carry out the isolation and cultivation of microglia with the possibility of further recreation of disease models in animals with the participation of these cells.

A major part of the study of microglial cells is carried out on rodents, whose microglia have a higher proliferative potential *in vitro* in comparison with human microglia. In addition, rodent and human microglia differ in the production of inflammatory mediators (for example, TGF-β1 is important for mice, but not so important for adult human microglia); in the expression of receptors (for example, the TLR4 lipopolysaccharide activates microglial cells in mice, but it is less significant for human microglia due to the presence in the latter of a higher diversity of genes encoding immunoglobulin-like lectins (siglecs) that bind sialic acid); in the generation of nitric oxide (the activity of inducible NO-synthase, and, consequently, the production of nitric oxide is much lower in human microglia). All this should be taken into account when extrapolating the results obtained in the study of rodent microglia to humans [3].

Currently, the protocols for the generation of microglia from mouse embryonic stem cells and induced human pluripotent stem cells have been developed. However, the methods proposed in them are too costly and the concept of how well the cells obtained in this way represent the properties of the resident microglia of the brain is not yet fully known [3, 11–13]. Therefore, despite the appearance of induced pluripotent human and animal cells as a potential tool for studying microglia, simpler protocols for isolating and cultivating microglial cells from rodents are still most important.

In this regard, **the aim of the present study** was to review the main methods for the isolation and cultivation of microglial cells in rodents.

## The main functions of microglia. Microglia types

Microglia are less common in the brain than other types of glia. However, they are involved in the homeostasis of the adult and developing brain, performing a number of functions, three of which are most important [1, 3, 14–16]:

“patrolling” — constant monitoring of the “environmental” changes (microglial cells continuously “scan” their micro-environment with long ramified processes called the sensome);

“maintenance” — abundantly ramified cells interacting with neurons and astrocytes participate in synaptic remodeling, myelin homeostasis, remove damaged cells and their fragments, ensure normal functioning of neurons;

“protection” — the “warriors” are activated microglial cells with a larger body and less ramified processes; they cause neuroinflammation, condition a response to harsh damaging agents, and provide neuroprotection under these conditions, but not so often.

In general, microglial cells provide [1, 3, 14–16]:

homeostasis of the adult brain — they remove damaged and/or infected cells during inflammation, including aseptic and infectious processes, as well as during injuries and neurodegenerative diseases; they participate in the metabolism of neurotransmitters and maintenance of extracellular ion homeostasis;

homeostasis of the developing brain — they perform the function of programmed destruction of nerve cells during embryogenesis;

release of neuroactive substances, such as eicosanoids, cytokines;

neuroinflammatory response — a massive release of eicosanoids, cytokines, chemokines, reactive oxygen species, nitrogen, and other inflammatory mediators;

regulation of neurogenesis — they are a structural part of the neurogenic niche, they can enhance or block neurogenesis depending on the features of a stimulus and the type of pathological process in the central nervous system: for example, in apoptosis and/ or ischemia, the “proneurogenic” microglia release IGF-1, TGF-β and enhance neurogenesis in the dentate gyrus of the hippocampus and/or subventricular zone, and the neuroinflammatory microglia in chronic neurodegeneration produce IL-6, IL-1β, and TNF-α and most often inhibit hippocampal neurogenesis;

(re)myelination and demyelination of neuronal axons.

Under physiological conditions, the brain contains such types of phagocytes as “resting” (inactive, M0 phenotype) microglia, perivascular macrophages, pericytes, a small number of so-called patrolling macrophages. “Resting” microglia are characterized by low expression of antigens on the cell surface, minimal release of chemokines and cytokines. It is involved in homeostasis, repairing damaged cells, but does not participate in phagocytosis [17]. Under physiological conditions, three main morphotypes of microglia were found: “stellate” microglia, “expanded” cells, “spindle-like” microgliocytes. In acute and chronic neurodegeneration, new morphotypes, the formation of which largely depends on microenvironmental signals, have been described (Table 1) [18–26].

**Table 1:** Morphological types of microglia *in vivo* in health and disease [18–26]

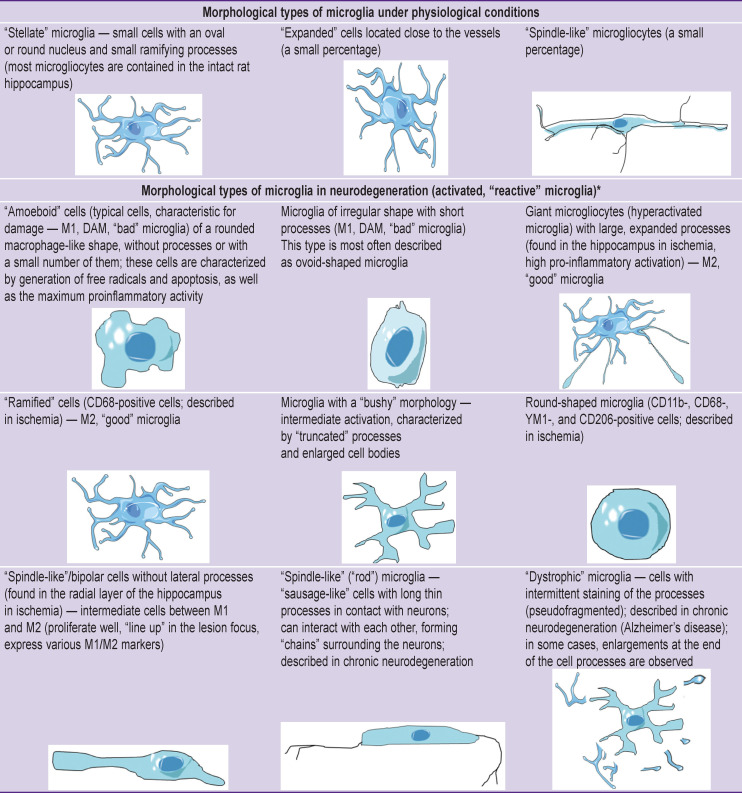

Notes: some of the drawings were made using predesigned vector images from the site https://smart.servier.com; *activated microglia, in comparison with resting microglia, on the whole, have reduced ramifying and enlarged cell bodies.

An increase in the absolute number and/or activity of microglial cells in response to damage (“reactive” microgliosis) is characterized by increased proliferation, activation, “recruiting” (further involvement) of microglia, an increase in the number of processes and connections with other cells.

A number of authors distinguish two main types (variants) of polarization of microglia during their activation: M1 and M2 [27–34]. It has been established that in terms of morphology and functional characteristics, the M1 and M2 microglia cells differ significantly from each other and play different roles in health and in various diseases of the central nervous system. The classical (proinflammatory, destructive) M1 phenotype (M1 polarization variant) causes a strong inflammatory response, is characterized by enhanced synthesis of proinflammatory cytokines, reactive oxygen and nitrogen species, and a pronounced antigen-presenting ability. The main function of the M1 phenotype is to destroy any damaging agents, including microorganisms and tumor cells. The alternative (anti-inflammatory, protective) M2 phenotype is involved in more “sophisticated” processes, such as removing cell detritus, activating angiogenesis, remodeling processes of the tissues and their repair, synaptic pruning [27], and synaptic remodeling [28, 29]. The M2 phenotype often has an anti-inflammatory effect due to the production of anti-inflammatory cytokines (IL-3, -4, -10), glucocorticoids, TGF-β. However, the effects of the M2 phenotype predominance can be not only “beneficial”, but also “harmful”, contribute, for example, to the chronicity of inflammation [30–34]. There are several subtypes of M2 polarization variants: M2a (for phagocytosis and subsequent tissue repair), M2b (for “recruiting” immunoregulatory T-lymphocytes), M2c (with anti-inflammatory properties) [8].

Other authors [35] differentiate such types of microglia as “good” and “bad” ones. “Good” microglia (most often we talk about the M2 variant of polarization) have a ramified structure, pronounced phagocytosis, produce IL-1β, IL-6, TNF-α, TGF-β, nitric oxide, with no development of neuroinflammation; express CD45, CX3CR1 antigens on their surface. “Bad” microglia, some researchers call them DAM — disease-associated microglia, on the contrary, form few processes, most often have an ovoid shape, a low ability to phagocytosis, promote the development of neuroinflammation, express Cx3cr1, CD40L, S100B, Cdk5/p25 antigens. Some researchers differentiate dysfunctional and “ugly” microglia [36, 37].

The general mechanism of pathogenesis of many neurological diseases, including neurodegenerative ones, is glia activation. However, the regional specificity of their activation is associated with the localization of not only the direct application of the damaging agents but also their types (an exogenous or endogenous agent, pathogen or damaging agent, etc.), as well as the specificity of their action [38]. Activated microglia play a key role in the pathogenesis of neuroinflammation, also participating in neurodegeneration (death of neurons), exhibit both destructive (damage to their own neurons by various cytotoxic molecules, that causes neurodegeneration) and protective (the same cytotoxic molecules kill pathogens, neurons that have mutated or which have been infected with viruses or other microorganisms) effects on the body, depending on the predominance of certain chemokines, cytokines, growth/ neurotrophic factors, neurogenic transcription factors. The balance between these mechanisms determines what happens to the neurons when the nervous system is damaged [15, 38–40].

Thus, activation of microglia in acute neurodegeneration which results from ischemia or brain injury has mainly a destructive effect due to the production of reactive oxygen and nitrogen species, proinflammatory cytokines, and activation of matrix metalloproteinases for the destruction of the extracellular matrix [39, 41]. However, the microglia effects also depend on the type of cerebral ischemia. The rats with global ischemia display a pronounced pro-inflammatory response and activation of M1 microglia [40], induction of microglial NLRP3 inflammasomes [42], as observed in many other types of chronic neurodegeneration [43]; microglial pyroptosis and subsequent inflammatory infiltration [42]. In focal ischemia, at the early stages of damage, the activation of the M2 phenotype manifests itself as a protective mechanism, and, further, the activation of the M1 microglia phenotype also occurs [15]. In addition, microglia of the M2 phenotype phagocyte migrating neutrophils, remove excess toxins and glutamate, prevent the invasion of neutrophils into the lesion focus (due to TNF-α), and promote tissue regeneration in the post-ischemic recovery phase [17, 30, 39].

Classical chronic neurodegeneration, for example, Alzheimer’s disease, is characterized by severe microgliosis [44, 45], impaired cellular detoxification and metabolic mechanisms, increased β-amyloid deposition, mitochondrial dysfunction, and, as a consequence, energy deficiency and oxidative stress, impaired calcium homeostasis, and induction of apoptosis [46, 47]. At the early stages of Alzheimer’s disease, “good” (M2) microglia can have a neuroprotective effect [35] such as, for example, inducing absorption and degradation of β-amyloid prior to its accumulation [17]. Activation of “bad” (mainly M1) microglia can lead to phagocytic dysfunction [48], pronounced production of neurotoxic chemokines, cytokines, reactive oxygen and nitrogen species [17]. This type of microglia, due to cell damage, on the contrary, promotes β-amyloid accumulation. This is also favored by insulin resistance of the brain, previous local or systemic infections, most likely causing the “depletion” of phagocytes, including microglia [18, 49]. Therefore, the correction of systemic inflammation and/or insulin resistance is one of the potential strategies for the prevention or early therapy of neurodegeneration in the elderly [50]. It is noteworthy that the development of Alzheimer’s disease can also be preceded by “age-related” changes in microglia such as an increase in the number/density of microglial cells, decreased regulation of their distribution, translocation to new areas of the brain, senile changes within them (accumulation of lipofuscin and mutations of mitochondrial DNA, telomere shortening, increased iron deposition), leading to pathological activation of microglia, oxidative stress, and impaired cell proliferation ability. Age-related changes in the morphology of microglial cells include perinuclear cytoplasmic hypertrophy, dystrophy, loss of processes; changes in their functions: decrease in the speed of movement, migration, inhibition of neurogenesis [51]. These altered cells are called dysfunctional microglia [17].

## Methods and techniques for isolating microglia

Human microglia are obtained in three ways [3, 11, 13, 52]:

when removing tumor tissue or resecting an epileptogenic focus (however, for example, the glioma contains many cells of monocytic origin, in fact, they present a mixture of microglial cells and infiltrated monocytes; the tissue from an epileptic focus is an abnormal environment for cell development);

from the postmortem tissue of the brain (however, the possibility of isolating cells from this tissue is regulated by the domestic law of a state, and the safety of cells depends very much on the cause of death and duration of the time interval after it);

from induced pluripotent stem cells.

Viable and well-preserved microglial cells can be obtained directly from any region of the rodent brain (most often they are isolated from the cerebral cortex, hippocampus, cerebellum, spinal cord) [53–60] after killing the anesthetized animals [61, 62] or by isolating bone marrow cells-precursors and differentiating them into microglia in a cell culture [63, 64] (Table 2).

**Table 2:** Basic methods for the isolation and/or cultivation of microglia from rodents

Rodents used for microglia isolation	Method	Features of the isolated cells	Application of the isolated cells
Newborn mice aged P0–P2 (neonatal microglia) [53]	Microglia “depletion” (by exposure to macrophage toxin — clodronate) and resettling of organotypic slice cultures of the hippocampus 375 μm thick with the microglia culture.	In a slice culture, cells have a ramified morphology, similar to that of microglia *in vivo*,	Study of the microglia, close to the *in vivo* situation, evaluation of the
Mice aged 8–16 weeks (adult microglia) [53]	The technique is quite laborious: the preparation of the slices takes about 3.5 h; mixed glial cultures — 4 h; isolation of adult microglia — 3.5 h; culture replenishment — 30 min	form intercellular contacts with other types of cells	intercellular interactions
Newborn mice aged P0–P2, both of wild type and genetically modified (neonatal microglia) [53]	Obtaining a primary mixed glial culture by trypsinization of the cerebral cortex; the mixed culture is cultivated for 2 weeks with 50% medium change every 3–4 days (after 2 weeks, the microglia should be on the top of the astrocyte monolayer); the microglia are separated by shaking (15 min at a frequency of 120 rpm); the resulting solution with a nutrient medium contains microglia cells. A week later, a new portion of microglia is collected in the same way, collection is possible within no longer than 4 weeks from the start of cultivation of the mixed culture	Cellularity usually amounts for 4–8**·**10^5^ living cells from 5–6 mice	The following cultivation and collection of the proliferating microglia take place within 2–4 weeks
Wistar rat pups aged P3–P5 (neonatal microglia) [54]	Microglia isolation in the Histodenz gradient (a “cloud” with a mixed culture of microglia and astrocytes between the 20 and 10% Histodenz layers)	Homogeneous population of uniform rounded “amoeboid” cells with Iba-1, CD68, CD11b/c, CD45, and nestin expression, without GFAP and NF200 antigens expression; at least 1 million viable microglia cells were obtained from 1 individual	The following cultivation and collection of proliferating microglia with cellularity of 2.5**·**10^5^ weekly within 2–8 weeks
Rat pups of the Sprague Dawley line at the age of P1 (neonatal microglia) [55]	Isolation of microglia from the cerebral cortex to obtain a mixed glial culture, used for microglia collection after 2 weeks and a 3D model recreation in a hydrogel by encapsulating cells in the hydrogel	The cell population forms not a monolayer, but a three- dimensional model; the cells can have a ramified morphology, similar to that of microglia *in vivo*, and form intercellular contacts with other types of cells	The following study of intercellular interactions, formation and study of the microenvironment of neurogenic niches, recreation of pathological processes
Mice aged 8–16 weeks (adult microglia) [53]	Isolation of microglia in the Percoll gradient (the lower layer of 75% Percoll is mixed with a homogenate, then a 25% Percoll layer, and then PBS): a thick and viscous upper “cloud” between the PBS and 50% Percoll layers contained all the elements of the central nervous system, with the exception of microglia; the lower “cloud” between layers of 75 and 25% Percoll-contained microglia cells	The cellularity usually amounts for 1–3**·**10^5^ brain cells in one adult mouse. The homogeneous population of uniform rounded cells with Iba-1 expression without antigens for GFAP astrocytes, the viability of isolated cells is more than 98%	The following analysis of functional activity/antigen expression or cultivation
Male Sprague Dawley rats aged 2–3 months (adult microglia) [56]	Isolation of microglia in the Percoll gradient (the lower layer — 70% Percoll — is mixed with a homogenate, then a 50% Percoll layer, and then PBS): the upper “cloud”, thick and viscous, between the PBS and 50% Percoll layers, contained all the elements of the central nervous system other than microglia; the lower thin “cloud” between the layers of 70 and 50% Percoll-contained highly enriched microglia, free of macrophages of the central nervous system	Homogeneous population of homogeneous rounded cells with Iba-1 expression without antigens for GFAP astrocytes, the viability of the isolated cells is more than 98%	The following analysis of functional activity/antigen expression or cultivation
Mice of over 8 weeks old (adult microglia) [57]	Isolation of microglia in the Percoll gradient (the lower layer — 70% Percoll — is mixed with a homogenate, then layers of 37 and 30% Percoll, and then PBS): the upper “cloud” with myelin between the layers of PBS and 30% Percoll; the lower “cloud” between the layers of 70 and 37% of Percoll-contained microglia cells	The population of uniform rounded cells with CD68 expression with single astrocytes (GFAP expression)	The following analysis of functional activity/antigen expression or cultivation
Mice aged 20–30 days and older (adult microglia) [58–60]	After separation of myelin by centrifugation in the Percoll gradient, microglia are isolated by the method of positive magnetic separation using “magnetic beads” bound to antibodies against CD11b	Population of homogeneous rounded cells with CD11b expression	The following analysis of the functional activity/ expression of the antigens or cultivation; in some cases, “magnetic beads” remaining on the cell surface can interfere with the study
C57BL/6 mice aged 2–3 months (adult microglia) [63, 64]	Isolation of microglial cells during their differentiation from bone marrow progenitors	Differentiated cells have a varied morphology (“amoeboid” and rounded, i.e. activated; only a few cells have clearly visible branches typical of primary resting microglia); isolation purity is over 90%	The following analysis of functional activity/antigen expression or cultivation

The method of obtaining a primary (mixed) glial culture for microglia isolation by intermittent density gradient centrifugation is quite laborious. In this method, several solutions with different densities are used, and, at the border of some of them, a “cloud” of microglia cells is formed [54, 56, 57].

The current and fast methods include microglia isolation using magnetic nanoparticles or specialized devices (for example, a flow cytometer with a cell sorting function). The most affordable nanoparticles in terms of costs are “magnetic beads” [1, 65, 66], but their use in some variants (especially with a negative method of isolating microglia cells) requires rather costly consumables.

Any methods of isolating microglia from the brain include the following stages [54, 56, 57, 67–69]:

1. transcardial intravital perfusion of an anesthetized animal with cold (4°C) PBS/DPBS solution with heparin to remove mononuclear blood cells in order to prevent further contamination of the microglial culture;
2. decapitation of the animal, rapid opening of the cranium on ice, extraction of the brain, placing it in a Petri dish with serum-free solution (serum-free DMEM, DPBS solution with glucose, or other serum-free solutions/media);
3. extraction of the needed structures of the brain (cerebral cortex, hippocampus, etc.) while removing other structures (for example, olfactory bulbs, cerebellum, optic tract, meninges) in a Petri dish on ice;
4. grinding the extracted structures by mechanical (cutting into pieces of 1 mm^3^ with a scalpel and/ or grinding the structures in special homogenizers; trituration/pipetting through a Pasteur pipette, dispensers, or syringes with tips/needles of a smaller diameter, etc.) and/or enzymatic (papain, DNase, dispase enzymes in a mixture or separately) methods;
5. washing off enzymes, if they have been used, filtering the homogenate through nylon or other filters with a pore size of 40–100 μm (to remove unseparated pieces of tissue); cell sedimentation by centrifugation (200–700 g for 5–10 min);
6. a specific method of cell isolation: when using “magnetic beads” to separate microglial cells, centrifugation is performed for 30–45 min at 4°С and 300–1200 g without sharp acceleration and deceleration in the density gradient (Percoll, Histodenz, etc.) in saline;
7. 1–2-fold washing off the collected cells (collection of the microglia cell sediment bound to the “beads” and sedimented on the bottom of a magnetic stand in the positive method of cell isolation; collection of the supernatant with microglia when other types of cells are precipitated in the negative method of cell isolation using “magnetic beads”; collection of the ring with the isolated cells in the density gradient using a Pasteur pipette/ dispenser with a fine tip or a spinal needle) in a buffer/ medium solution, followed by cell centrifugation and adding the required volume of buffer/culture medium at the final stage;
8. seeding cells in a nutrient medium with subsequent cultivation or use of microglial cells for further research (if the isolation of microglial cells does not suggest their further cultivation).

It is rather difficult to isolate microglia from the material obtained from adults. Most studies have been performed on neonatal microglia since the yield of adult microglial cells is low. Blood monocytes and fibroblast-like cells of the bone marrow can differentiate into microglia (cytokines, hypoxia, and immune cells provoke differentiation). Mesenchymal stem cells derived from bone marrow and transplanted into the brain lead to a decrease in amyloid plaques. Most likely, it happens due to the activation of the resident microglia to an amoeboid and phagocytic state. Besides, mesenchymal stem cells have a positive effect on neurodegeneration by regulating the microenvironment and the cells themselves, the microglia in particular [63, 64, 70].

The mesenchymal stromal cells of the bone marrow (BMCs, BM-MSCs) represent a population of cells that form skeletal muscle and hematopoietic stroma *in vivo* and have osteogenic potential. BM-MSCs meet the strict criteria for stem cells *in vivo*: they proliferate as non-adhesive mesenspheres, and not as adherent cultured cells.

The methods for isolating microglial cells during their differentiation from bone marrow progenitors of BMCs include the following stages [63, 64, 70–82].

1. Isolation of the femur/lower leg bones from euthanized animals, opening of the bones and their centrifugation (at ≥10,000 g for 15 s) in the nested microcentrifuge tubes with a volume of 0.5 ml with a hole and 1.5 ml without holes for obtaining bone marrow; resuspension of the isolated bone marrow in solution (for example, PBS, nutrient medium, FACS buffer).
2. With a 10^7^ cellularity, bone marrow cells are cultured for 24 h in a Petri dish in the DMEM medium with a low glucose level with 10% FCS, 10^–8^ M dexamethasone, and 100 U/ml of penicillin and streptomycin.
3. Washing off non-adherent cells after 24 h and transferring them to a new Petri dish. The 24-hour adhesion period is repeated 4 times to obtain non-adherent NA-BMC cells.
4. Resuspension of non-adherent cells of the 1^st^ day (a classic seeding protocol for obtaining macrophages or microglia) and NA-BMCs of day 4 in osteogenic (DMEM, 10% FCS, 10^–8^ M dexamethasone, 50   μg/ml ascorbic acid) or conditioned medium: astrocyte-conditioned medium as a source of M-CSF is obtained by growing a primary culture of astrocytes for 24 h. 20% (by volume) fibroblast-conditioned medium, endotheliocyte-conditioned medium, or conditioned medium from intact or injured rat spinal cord can also be used. Ready-made M-CSF at a concentration of 10 ng/ml can be used. The medium is changed every 3 days, and the cells are analyzed on the 10^th^ day of growth.

One of the protocol variants for isolating microglia cells during their differentiation from bone marrow progenitors shows microglia to differentiate on days 3 and 6 of cultivation, and macrophages — on days 3 and 4 [82].

## Microglia cultivation methods

Among the methods of microglial cell cultivation (see Table 2), 2D (in special plastic plates/Petri dishes, bottles with a special coating with collagen, laminin, polylysine, and others or without it) and 3D (in a matrix-gel or scaffolds) cultivation can be distinguished as well as microglia cultivation on hippocampal slice cultures [53, 54].

Microglia culture medium usually includes DMEM/ F12 or DMEM as a base. It is enriched with 10% fetal bovine serum and L-glutamine, and contains penicillin-streptomycin or other antibiotics to exclude contamination of the medium with microorganisms. To provide enrichment, selection of the direction for differentiation, and obtaining a precipitated cell culture (otherwise, most of the cells remain in suspension and do not adhere to the plastic surface), the cultivation medium is supplemented with one of the growth/ differentiation factors (for example, multipotential CSF/multi-CSF and granulocyte/macrophage CSF/G/M-CSF are mitogens for microglia, therefore, the G-CSF granulocyte colony-stimulating factor can be used to stimulate proliferation and biological activity of amoeboid microglia) or lipopolysaccharide/interferon solution and other reagents for polarization of the microglia into the desired phenotype. Thus, the conversion of microglia to the M1 phenotype usually occurs under the action of lipopolysaccharide, IL-1β or TNF-α. The detection of such M1 microglia markers as iNOS, COX-2, and IL-6, and the loss of M2a markers can be noted in this case. The CD38, Gpr18, and Fpr2 molecules, which are M1 polarization markers, as well as Egr2 and c-Myc markers, “exclusive” for the M2 phenotype, can be observed on the macrophages. IL-1RA, SOCS3 are the markers of M2b-immunomodulatory microglia (M2b polarization occurs under the action of IL-10). The presence of arginase-1, CD206, and the loss of markers M1 and M2b microglia (M2a polarization occurs under the action of IL-4) can be considered as the markers of M2a repair and regeneration [54, 57–60, 83–85].

To provide long-term (6–8 weeks) cultivation and proliferation of microglia, it is necessary to use growth/ differentiation factors (for example, G-CSF), an incomplete medium change 2 times a week (usually 1/3–1/2 of the medium is replaced with a fresh one), and also co-cultivation with astrocytes [54, 57]. The weekly yield of cells from one T75 vial achieves 2.0–2.5**·**105; after 8 weeks of cultivation, the proliferation of microglia practically stops, it is necessary to reseed the cell culture or collect and freeze the cells for the future, if necessary.

Hydrogel cultures of nerve cells, for example, with the use of Matrigel, have been created to overcome the problems associated with two-dimensional cell cultures: the formation of only a monolayer of cells; difference between the morphology of these cells and the morphology of cells *in vivo*; poor representativeness of the models of CNS damage, particularly, when it comes to the formation of biological barriers, including the blood-brain barrier. Hydrogel cultures have a wide range of applications, including pharmacological research, study of the control of nerve cell differentiation, their use for the understanding of disease etiopathogenesis, co-cultivation with other types of cells (models of neurogenic niches), their use for the modeling of the cell migration process, neuroprotection/neurodegeneration or for the modelling of the tissue microenvironment. 3D technologies allow the formation of hydrogels of various dimensions and shapes. These cultures are easily analyzed using widespread research methods such as fluorescence and confocal microscopy [55]. 3D cultivation, in contrast to 2D, has a number of advantages [86]:

the cells have a more natural shape;

they proliferate faster or slower depending on the needed conditions;

3D models contain cells at various stages of the cell cycle, including proliferating, resting cells, cells in hypoxia, apoptotic, and/or necrotic cells;

3D models display various rates of penetration of nutrients or drugs into cells, including the ability to mimic drug resistance, which is closer to the *in vivo* situation and is relevant when studying the potential effect of a drug;

these models demonstrate gene and protein expression profiles analogous to cells *in vivo*.

Thereby, it is characteristic for practically all types of cells that 3D *in vitro* models are more similar to *in vivo* models than 2D models, therefore, they are more significant biologically, too [75]. In this regard, in recent years, the use of scaffolds (“scaffolds” that imitate the intercellular matrix), various “solid platforms”, aggregates, micro- and nanofibers for cell cultivation in a three-dimensional microenvironment has increased [86, 87].

Quite a number of 3D cultivation variants have been tested for astrocytes (cultivation in collagen hydrogel, in hydrogel matrices with collagen or nanofibers, on scaffolds of various nanofibers, etc.) [87–102], whereas only models grown in a hydrogel like Matrigel, in a peptide hydrogel PuraMatrix, on graphene scaffolds, and on collagen matrices have been investigated for microglia [103–105].

## Changes in microglia during cultivation

When isolating rodent microglia, cells of two main morphological types can be obtained:

“amoeboid” microglia of the perinatal period, also characteristic for newborn animals, the number of which in rats and mice begins to sharply decrease from about the second week of life and is gradually replaced by “ramified” ones;

“ramified” — resting “adult” microglia, the number of which increases from the second week of life [19–21, 54].

When microglia are obtained from the brain, most often the cells initially have an “amoeboid” morphology (a rounded shape and 8–10 μm in diameter with highly vacuolated cytoplasm). For the first 1–2 days (3–4 days according to other data) of cultivation, most of them are suspended and precipitate well on the 2–5^th^ day of cultivation in the plastic laboratory glassware. Single isolated astrocytes form a monolayer on the surface of the plastic, on the top of them, the first single microglial cells settle, which form the islets then, the cells from them are periodically separated into the medium in the form of a suspension culture. The most active microglia separation goes on mainly from the 2^nd^ to the 6^th^ week of cell cultivation, while the separated cells also have an “amoeboid” morphology, and the adhered cells start to form 1–2 processes on the 5–6^th^ day of cultivation; cells of a “ramified” morphology are rarely found [22–26, 54].

In 3D cultures, microglial cells are more evenly spread over the width of the gel, have a predominantly ramified morphology with long and multidirectional branches in different projections and a smaller cell body in terms of the cytoplasm area, in contrast to two-dimensional cultures. Due to this, they are closer to resting microglia *in vivo* [106].

## Conclusion

Microglia are polyfunctional mesodermal cells of the nervous system, among which two most important morpho-phenotypes can be distinguished, those are M1 (“bad”, DAM) and M2 (“good”) microglia. Microglial cells are involved in maintaining homeostasis of the developing and adult brain under physiological conditions, as well as in the development of acute and chronic neurodegeneration. The most feasible and least laborious methods among the considered ones are the microglia isolation by density gradient centrifugation and/or using “magnetic beads” (although the cellularity with these methods is not always high) and cultivation in a three-dimensional culture microenvironment using hydrogels, scaffolds, aggregates, and fibers. Microglial cells stained in this way are close to microglia *in vivo* in terms of a morphotype, antigen expression, and degree of activation.
